# Regulating autonomic nervous system homeostasis improves pulmonary function in rabbits with acute lung injury

**DOI:** 10.1186/s12890-017-0436-0

**Published:** 2017-07-03

**Authors:** Yan Liu, Tao Tao, Wenzhi Li, Yulong Bo

**Affiliations:** Department of Anesthesiology, the Second Affiliated Hospital of Harbin Medical University, the HeiLongJiang Province key laboratory on Anesthesiology and Critical Care Medicine, NO.246 of Xuefu Road, Nangang District, Harbin, Heilongjiang Province 150081 China

**Keywords:** Sympathetic nervous system, Parasympathetic nervous system, Acute lung injury, HCl aspiration, Stellate ganglion block

## Abstract

**Background:**

This study aimed to investigate the effects of regulating autonomic nervous system (ANS) homeostasis by inhibiting sympathetic hyperactivity and/or enhancing parasympathetic activity on pulmonary inflammation and functional disturbance.

**Methods:**

An animal model of acute lung injury (ALI) was established in rabbits by an intratracheal injection of hydrochloric acid (HCl) in rabbits. Animals in control groups were received saline or HCl only, and the others received both HCl and followed treatments: vagus nerve stimulation (VNS), intravenous injection of tetrahydroaminoacridine (THA), or stellate ganglion block (SGB). The effects of different treatments on the changes in autonomic nervous system homeostasis, pulmonary and systemic inflammation, and functional disturbance were detected.

**Results:**

Sympathetic nervous activity was higher than parasympathetic nervous activity in rabbits after HCl aspiration, as demonstrated by the significant changes in the discharge frequency of cervical sympathetic/vagus trunk, and heart rate variability. VNS, THA and SGB could significantly alleviate the changes of ANS induced by HCl aspiration and improved the pulmonary function, especially for SGB treatment.

**Conclusions:**

The disturbance of ANS homeostasis is attributed to a predominance of SNS activity. Administration of VNS, THA and SGB are capable to regulate disequilibrium of the ANS in rabbits with HCl-induced ALI and SGB is supposed to be the most effective approach.

## Background

When the balance between the sympathetic nervous systems (SNS) and parasympathetic nervous systems (PNS) is disturbed by some events, such as serious stress and inflammation, strong sympathetic impulse, may shift the autonomic nervous system (ANS) homeostasis towards sympathetic dominance, leading to various diseases associated with sympathomodulation [1, 2]. Inhibiting SNS hyperactivity and/or adjusting PNS activity plays an important role in modulating ANS balance, and improves clinical outcomes in patients with severe inflammation.

As described previously, stellate ganglion block (SGB) is capable to accommodate ANS homeostasis through reducing SNS hyperactivity in a stress response [3, 4]. The inflammation can be inhibited by enhancement of PNS activity through activating the cholinergic anti-inflammatory pathway (CAP) via the efferent vagus nerve by specific alpha7 nAChR agonists [5], choline [6], or cholinesterase inhibitors [7] such as tetrahydroaminoacridine (THA), and electrical vagus nerve stimulation (VNS) [8]. But the most effective approach to achieve the most improvement for patients with severe stress and inflammation has not been confirmed. Although important progress has been made in understanding the physiological and pathological changes caused by stress and inflammatory infiltration, researches focusing on the extent of ANS homeostasis destruction, the roles of the SNS and PNS in inflammatory infiltration and conclusive therapies are still inadequate.

Acute lung injury (ALI) induced by hydrochloric acid (HCl) in animals in the laboratory can perfectly imitate the clinical manifestation of vomiting and aspiration after lung pathology. In the present study, a HCl-induced ALI model in rabbits was performed, aiming to explore the following: 1) the extent of the ANS disequilibrium after HCl aspiration; 2) whether stimulation of CAP or inhibition of SNS hyperactivity could effectively alleviate the ANS disequilibrium; 3) the optimal therapeutic approach for ALI after comparing the effects among different methods.

## Methods

### Animals

Forty adult male New Zealand White rabbits, weighing 2.7–3.2 kg, were purchased from the Animal Experiment Center of Harbin Medical University (Harbin, Heilongjiang, China) with a specific pathogen free housing condition. Then they were randomly assigned to five groups (8 per group): (1) control group, intratracheal injection of saline; (2) HCl group, intratracheal injection of HCl; (3) electronic vagus nerve stimulation (VNS) group, VNS and intratracheal injection of HCl; (4) tetrahydroaminoacridine (THA) group, THA (Shanghai Hanxiang, Shanghai, China) and intratracheal injection with HCl; and (5) stellate ganglion block (SGB) group, SGB and intratracheal injection with HCl. All animal experiments were approved by the Institutional Animal Care and Use Committee.

### Surgical procedure

Rabbits were first anesthetized by intravenous injection of pentobarbital sodium (30 mg/kg, Sigma, St. Louis, USA) via ear vein. After tracheotomy and tracheal intubation, animals were ventilated mechanically with O_2_ of 10 ml/kg, FiO_2_ of 1.0, and positive end-expiratory pressure of 3 cm H_2_O (ALC-V8 animal ventilator, Shanghai, China). Anesthesia was maintained with continuously pumping of 0.1 mg/kg/h pipecuronium (Arduan, Gedeon-Richter, Hungary) and 5 mg/kg/h pentobarbital, and lactated Ringer’s solution (Shanghai Harvest Pharmaceutical CO, LTD. Shanghai, China) was infused into the trachea of rabbit at 10 ml/kg/h. Then left femoral artery was cannulated to measure the mean arterial pressure (MAP) and the left cervical sympathetic nerve trunks were isolated carefully. In the VNS group, the right cervical vagus nerve was also isolated. In the SGB group, the right stellate ganglion was carefully exposed by a right lateral thoracotomy, and an epidural catheter was placed by sutures. Respiratory rate was regulated to maintain PaO_2_ over 400 mmHg and PaCO_2_ 35–45 mmHg before treatment.

### ALI model and treatment

Rabbits in the control group were injected intratracheally with saline of 2.8 ml/kg at 30 min after surgery through a cannula. HCl-induced ALI model was established by intratracheal injection of HCl (pH 1.5) of 2.8 ml/kg. In the VNS group, animals received constant voltage stimuli (5 V, 2 ms, and 1 Hz, Electrical stimulator, RM6240, Chengdu, China) to the right cervical vagus for 15 min after HCl aspiration. In the THA group, rabbits were injected intravenously with THA (3 mg/kg) after HCl aspiration. In the SGB group, 0.25% bupivacaine (Sigma, Taufkirchen, Germany) was continuously administered (0.5 ml/h) after a bolus injection of 5 ml through the catheter during the experiment. All of these three treatments were performed immediately after the ALI model constructed.

### Measurements

The discharge frequency in the cervical sympathetic/vagus trunk (CSTDF/CVTDF), peak airway pressure (PAP), lung compliance, blood gases (PaO_2_, PaCO_2_, and pH) were measured at 0 min (just before HCl aspiration), 5 min, 15 min, 30 min, 1 h, 2 h, 3 h, 4 h and 5 h after HCl aspiration by blood-gas analyzer GEM premier 3000 (Instrumentation Laboratory Company, Lexington, USA). Six hours after HCl aspiration, the rabbits were euthanized by bloodletting under anesthesia. The level of interleukin-6 (IL-6), IL-10, and tumor necrosis factor (TNF)-α in both plasma and bronchoalveolar lavage fluid (BALF) was detected with a commercial enzyme-linked immunosorbent assay (ELISA) kit (GBD, San Diego, CA, USA). Total protein level, leukocyte count and the percentage of polymorphonuclear neutrophils (PMNs) in BAFL were also examined. After the BALF obtained, a centrifugation at 4 °C, 2000 rpm for 10 min was performed. The sediment for this centrifugation was utilized to evaluate the leukocyte counts and percentage of PMNs by smear stained with Giemsa and Wright. All the detections were performed by two independently experienced inspectors via microscope.

During the procedure, the electrocardiograph (ECG) was inspected and recorded by multi-functional physiological detector MP150 (BIOPAC MP Hardware, MP150, 40 Aero Camino, Goleta, USA). After recording, 5 min of ECG without interpretation was selected and the R-wave was defined artificially. Then, the heart rate variability (HRV) analysis was performed using Kubios HRV software 2.0 (Biosignal Analysis and Medical Imaging Group, Department of Applied Physics, University of Eastern Finland, Kuopio, Finland) [9]. Meanwhile, measurement of low frequency (LF) by frequency bands ranged from 0.04 Hz to −0.15 Hz and of high frequency ranged from 0.15 Hz to 0.4 Hz in this 5 min of ECG were also examined. All parameters measured have been standardized in clinical settings. To exclude the man-made influence, the ECG was recorded before the detections of lung compliance, discharge frequency and blood sampling. No adverse events were identified in this short-term research.

### Histological assessment

After rabbits were sacrificed, lung tissues of animals were harvested and the wet weight of them were measured. After dried at 80 °C for 48 h, tissues were measured for dry weight. The wet weight/dry weight (W/D) ratio was used to evaluate pulmonary edema. Moreover, for histological analysis, parts of lung tissues were fixed in 10% formalin, embedded in paraffin and cut into 4 μm slices. After stained with hematoxylin-eosin (H&E), tissue sections were viewed for pathological changes under light microscope. Furthermore, the other parts of lung tissues were fixed in 2.5% glutaraldehyde and serially dehydrated. Then tissues were post-fixed in 1% osmium tetroxide and embedded in Araldite resin. Sections were observed under a transmission electron microscope.

### Statistical analysis

All data were expressed as mean ± standard deviation (SD). Statistical analyses were performed using SAS version 9.1.13 for Windows software (SAS Institute, Inc., Shanghai, China). The normality of the data distribution was assessed using Proc univarinate. Differences among groups were subjected to one-way analysis of variance followed by a post hoc Student-Newman-Keuls test. A *P* value < 0.05 was considered statistically significant.

## Results

### Improvement of pulmonary function in the three treatment groups

**Fig. 1:**
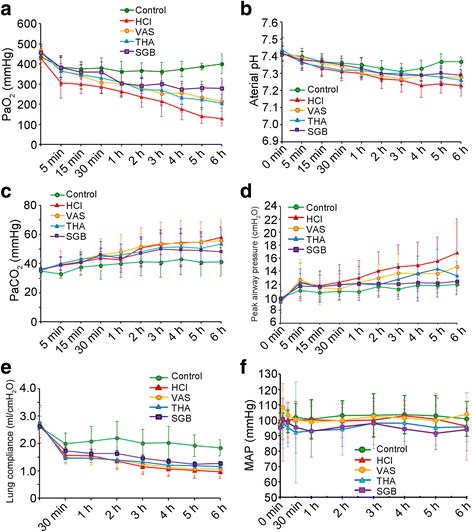
The alterations of pulmonary indicators in different treated groups at different times after surgery. **a**, the alteration of PaO_2_ in different groups after surgery; **b**, the alteration of aterial pH in different groups after surgery; **c**, the alteration of PaCO_2_ in different groups after surgery; **d**, the alteration of peak airway pressure in different group after surgery; **e**, the alteration of lung compliance in different groups after surgery; **f** the alteration of MAP in different groups after surgery. For control group (*n* = 8), rabbits only received saline intratracheally injection (2.8 ml/kg) after surgery; for HCl groups (*n* = 8), ALI model was established by intratracheal injection of HCl (pH 1.5, 2.8 ml/kg); for VNS groups (*n* = 8), after the ALI model established by HCl, rabbits also received constant voltage stimuli (5 V, 2 ms, and 1 Hz); for THA group (*n* = 8), after ALI model established by HCl, rabbits also received THA (3 mg/kg) intravenous injection; and for SGB group, after ALI model established by HCl, rabbits also continuously received 0.25% bupivacaine pumping via epidural catheter (3 mg/kg). VNS, vagus nerve stimulation; THA, tetrahydroaminoacridine; SGB, stellate ganglion block; ALI, acute lung injury; MAP, mean arterial pressure

When surgery was finished, no significant difference were identified in the pulmonary functional indicators among these five groups (*P* > 0.05), but conditions were changed since 5 min after surgery. An obvious decline of PaO_2_ was detected in HCl group after HCl aspiration compared with control group (t = 3.17, 3.07, 3.72, 3.89, 5.17, 5.94, 7.90, 9.85 and 10.88, *P* = 0.001, 0.002, 0.000, 0.000, 0.000, 0.000, 0.000, 0.000 and 0.000, respectively). However, all the three treated groups, VNS, THA and SGB, showed a significant alleviation on this decline in HCl group since 4 h, 3 h, 2 h after surgery, respectively (VNS, t = 3.25, 3.77 and 3.41, *P* = 0.001, 0.000 and 0.001, respectively; THA, t = 2.23, 2.27, 3.36 and 3.11, *P* = 0.027, 0.024, 0.001 and 0.002, respectively; SGB, t = 2.26, 3.48, 3.98, 5.63 and 6.05, *P* = 0.02, 0.001, 0.000, 0.000 and 0.000, respectively), but there PaO_2_ levels was still lower than control group (Fig. 1a). Similarly, significant declines were also observed in arterial pH (t = 2.10, 2.66, 2.60, 5.20, 6.56 and 6.80, *P* = 0.036, 0.008, 0.010, 0.000, 0.000 and 0.000) and lung compliance (t = 2.57, 3.14, 5.29, 5.57, 6.33, 5.89 and 5.74, *P* = 0.011, 0.0019, 0.000, 0.000, 0.000, 0.000, and 0.000, respectively) while compared with control group since 30 min after surgery (Fig. 1b and e). Meanwhile, VNS, THA and SGB also performed an alleviation on arterial pH reduction since 4 h, 3 h and 4 h after surgery, respectively (VNS, t = 3.03, 2.1 and 2.54, *P* = 0.003, 0.036 and 0.012, respectively; THA, t = 2.1, 3.27, 2.52 and 2.54, *P* = 0.036, 0.001, 0.012, and 0.012, respectively; SGB, t = 2.97, 2.71 and 3.22, *P* = 0.003, 0.007 and 0.002, respectively), but their improvements were still lower than control group. However, in the lung compliance, only SGB showed a significant improvement of the reduction induced by HCl inspiration since 4 h after surgery (t = 2.97, 2.72 and 3.22, *P* = 0.044, 0.007 and 0.002). Moreover, significant improvements were detected in PaCO_2_ (Fig. 1c) and peak airway pressure (Fig. 1d) in HCl group while compared with control group since 2 h (t = 2.29, 2.91, 2.70, 3.36 and 4.05, *P* = 0.023, 0.004, 0.007, 0.001 and 0.000, respectively) and 1 h (t = 2.08, 2.3, 3.31, 2.92, 3.67 and 4.67, *P* = 0.039, 0.023, 0.0011, 0.004, 0.000 and 0.000, respectively) after surgery, respectively. But only SGB could significant reduced improvement of peak airway pressure (t = 2.53, 2.52, 3.29 and 4.21, *P* = 0.012, 0.012, 0.001 and 0.000, respectively) induced by HCl since 3 h and after surgery, respectively. In addition, mean arterial pressures (MAPs) in groups were also continuously monitored throughout the experiment (Fig. 1f). According to the recordings, MAPs with HCl aspiration were stable compared with control group, but in the THA group, a significant decrease was shown at 30 min after treatment while compared with control and HCl groups (*P* < 0.05). However, both in VNS and SGB groups, the MAPs were obviously decreased throughout the experiment, but there were no remarkable differences identified between them and control group, as well as HCl group (*P* > 0.05).

### Changes in CSTDF and CVTDF in the three treatment groups

**Fig. 2:**
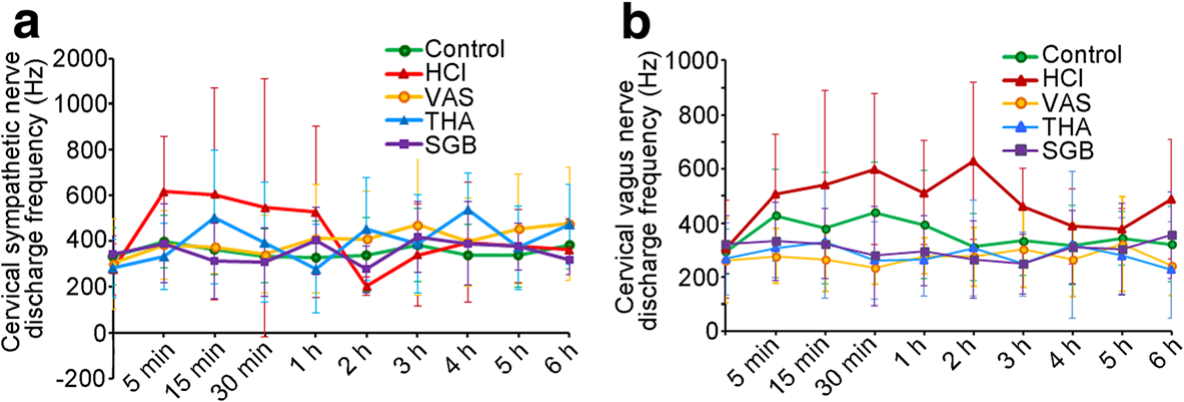
Changes of CSNDF and CVNDF in rabbits with different treatment at different time after HCl aspiration. **a**, changes of CSNDF in rabbits after HCl aspiration; **b**, changes of CVNDF in rabbits after HCl aspiration. For control group (*n* = 8), rabbits only received saline intratracheally injection (2.8 ml/kg) after surgery; for HCl groups (*n* = 8), ALI model was established by intratracheal injection of HCl (pH 1.5, 2.8 ml/kg); for VNS groups (*n* = 8), after the ALI model established by HCl, rabbits also received constant voltage stimuli (5 V, 2 ms, and 1 Hz); for THA group (*n* = 8), after ALI model established by HCl, rabbits also received THA (3 mg/kg) intravenous injection; and for SGB group, after ALI model established by HCl, rabbits also continuously received 0.25% bupivacaine pumping via epidural catheter (3 mg/kg). CSNDF, cervical sympathetic nerve discharge frequency; CVNDF, cervical vagus nerve discharge frequency; VNS, vagus nerve stimulation; THA, tetrahydroaminoacridine; SGB, stellate ganglion block; ALI, acute lung injury

Compared to the control group, changes in CSTDF and CVTDF fluctuate much more strongly in rabbits after HCl aspiration (Fig. 2a and b). But the fluctuations in the value of both CSTDF and CVTDF were much smaller after 3 different treatments including VNS, THA and SGB treatments. Specially, VAS could significance minish this aberrant changes in CSTDF at 5 min, 15 min and 30 min (t = 2.40, 2.39 and 2.10, *P* = 0.017, 0.018 and 0.036, respectively); THA could obviously decrease the changes at 5 min, 1 h and 2 h (t = 2.95, 2.45 and 2.61, *P* = 0.004, 0.015 and 0.010, respectively); and SGB could markedly reduce this changes at 5 min and 15 min (t = 2.35 and 3.03, *P* = 0.020 and 0.003, respectively) (Fig. 2a). Meanwhile, in the CVTDF, VAS could significance minish this aberrant changes in CSTDF at 5 min, 15 min, 30 min,1 h, 2 h and 3 h (t = 3.09, 3.70, 4.92, 3.16, 4.78 and 2.14, *P* = 0.002, 0.000, 0.000, 0.002, 0.000 and 0.033, respectively); THA could obviously decrease the changes at 5 min, 15 min, 30 min, 1 h, 2 h and 3 h (t = 2.70, 2.83, 4.53, 3.36, 4.35 and 2.90, *P* = 0.007, 0.005, 0.000, 0.001, 0.000 and 0.004, respectively); and SGB could markedly reduce this changes at 5 min, 15 min, 30 min,1 h, 2 h and 3 h (t = 2.37, 2.93, 4.29, 2.92, 4.91 and 2.88, *P* = 0.019, 0.004, 0.000, 0.004, 0.000 and 0.004, respectively) (Fig. 2b).

### HRV analysis after different treatment

**Fig. 3:**
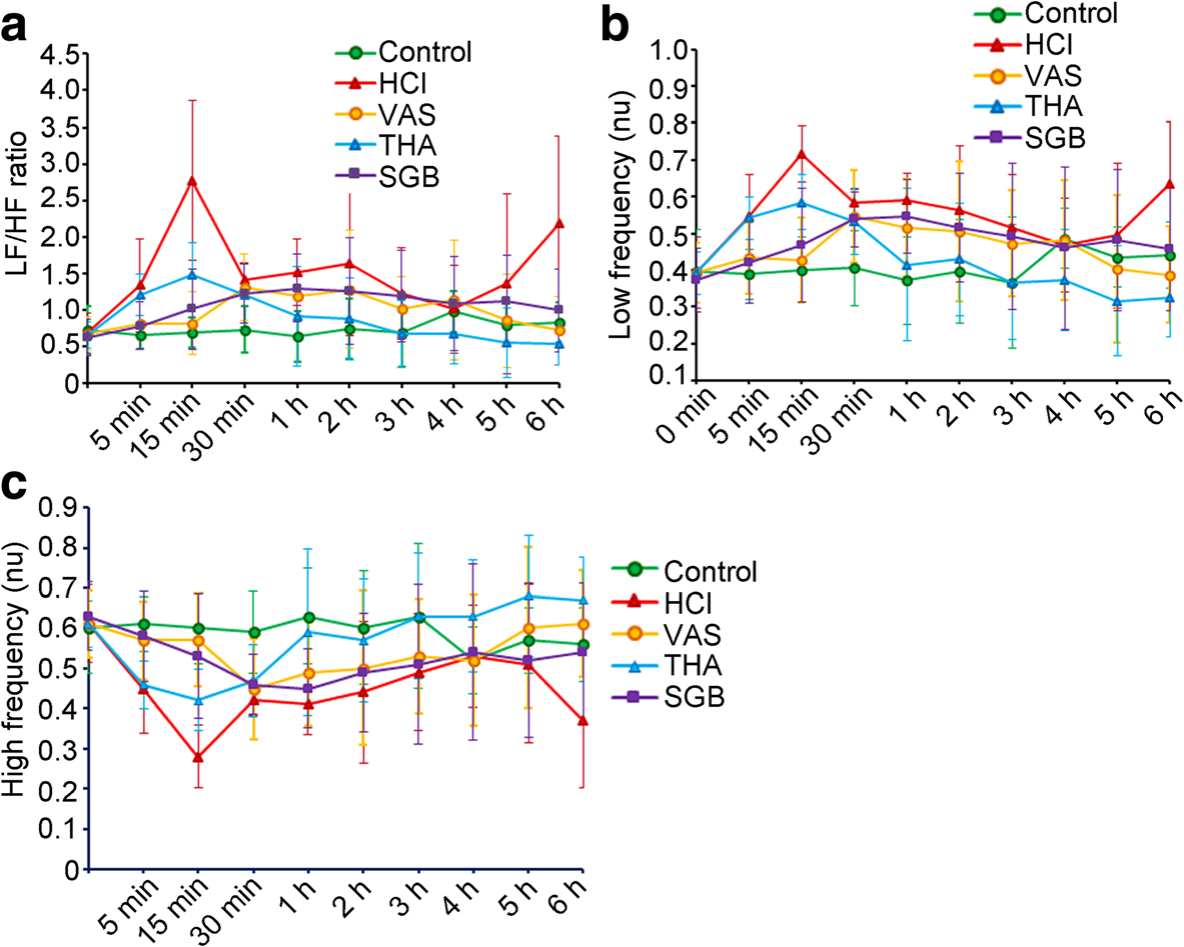
Heart rate viability alterations in rabbits with different treatment at different time after HCl-induced acute lung injury. **a**, alterations of LF/HF ratio in different groups; **b** alterations of LF in different groups; **c** alterations of HF in different groups. For control group (*n* = 8), rabbits only received saline intratracheally injection (2.8 ml/kg) after surgery; for HCl groups (*n* = 8), ALI model was established by intratracheal injection of HCl (pH 1.5, 2.8 ml/kg); for VNS groups (*n* = 8), after the ALI model established by HCl, rabbits also received constant voltage stimuli (5 V, 2 ms, and 1 Hz); for THA group (*n* = 8), after ALI model established by HCl, rabbits also received THA (3 mg/kg) intravenous injection; and for SGB group, after ALI model established by HCl, rabbits also continuously received 0.25% bupivacaine pumping via epidural catheter (3 mg/kg). LF, low frequency; HF, high frequency; VNS, vagus nerve stimulation; THA, tetrahydroaminoacridine; SGB, stellate ganglion block; ALI, acute lung injury

While compared to the control group, the LF/HF ratio was significantly elevated during the whole process in HCl group except at 5 min and 4 h (t = 0.11, 2.73, 8.24, 2.72. 3.5, 3.54, 2.11, 0.12, 2.21 and 5.39, *P* = 0.913, 0.007, 0.000, 0.007, 0.001, 0.001, 0.036, 0.902 and 0.028, respectively) (Fig. 3a). These alterations could be significant alleviated by VNS at 5 min, 15 min and 6 h (t = 2.08, 7.72, 2.00 and 5.83, *P* = 0.039, 0.000, 0.047 and 0.000, respectively), by THA at 15 min, 1 h, 2 h, 3 h, 5 h and 6 h (t = 5.10, 2.44, 3.00, 2.16, 3.19 and 6.59, *P* = 0.000, 0.015, 0.003, 0.031, 0.002 and 0.000, respectively), and by SGB at 5 min, 15 min and 6 h (t = 2.21, 6.97 and 4.69, *P* = 0.028, 0.000 and 0.000, respectively) (Fig. 3a). As a participator for LF/HF ratio, LF (nu) was also significantly elevated during the whole process after ALI model induced except 4 h and 5 h (t = 2.62, 5.22, 2.91, 3.59, 2.74, 2.43, 0.27, 0.97 and 3.22, *P* = 0.09, 0.000, 0.004, 0.000, 0.007, 0.016, 0.789, 0.334 and 0.002, respectively) compared with the control group (Fig. 3b). Meanwhile, these changes also could be impaired by VNS at 15 min and 6 h (t = 4.76 and 4.08, *P* = 0.000 and 0.000), by THA at 15 min, 1 h, 2 h, 3 h, 5 h and 6 h (t = 2.16, 2.89, 2.23, 2.43, 2.93 and 5.09, *P* = 0.004, 0.004, 0.027, 0.016, 0.004 and 0.000, respectively), and by SGB at 5 min, 15 min and 6 h (t = 2.08, 4.1 and 2.91, *P* = 0.038, 0.000 and 0.004, respectively). However, the level of HF (nu) was significantly reduced before 4 h after treated with HCl (t = 2.62, 5.22, 2.91, 3.59, 2.74 and 2.43, *P* = 0.009, 0.000, 0.004, 0.000, 0.007 and 0.016, respectively) (Fig. 3c). But only THA could remarkably alleviate these changes at most time points, including 15 min, 1 h, 2 h, 3 h, 5 h and 6 h (t = 2.16, 2.89, 2.23, 2.43, 2.93 and 5.09, *P* = 0.031, 0.004, 0.027, 0.016, 0.004 and 0.000, respectively). Additionally, compared THA group with control group, LF (nu) and the LF/HF ratio was remarkably elevated until 15 min post-HCl aspiration (t = 2.91, *P* = 0.004), then gradually decreased over time, and even fell below the value in the control group at 4 h after THA treatment (Fig. 3).

### Detection of inflammatory cell infiltration and vascular permeability after different treatment

**Fig. 4:**
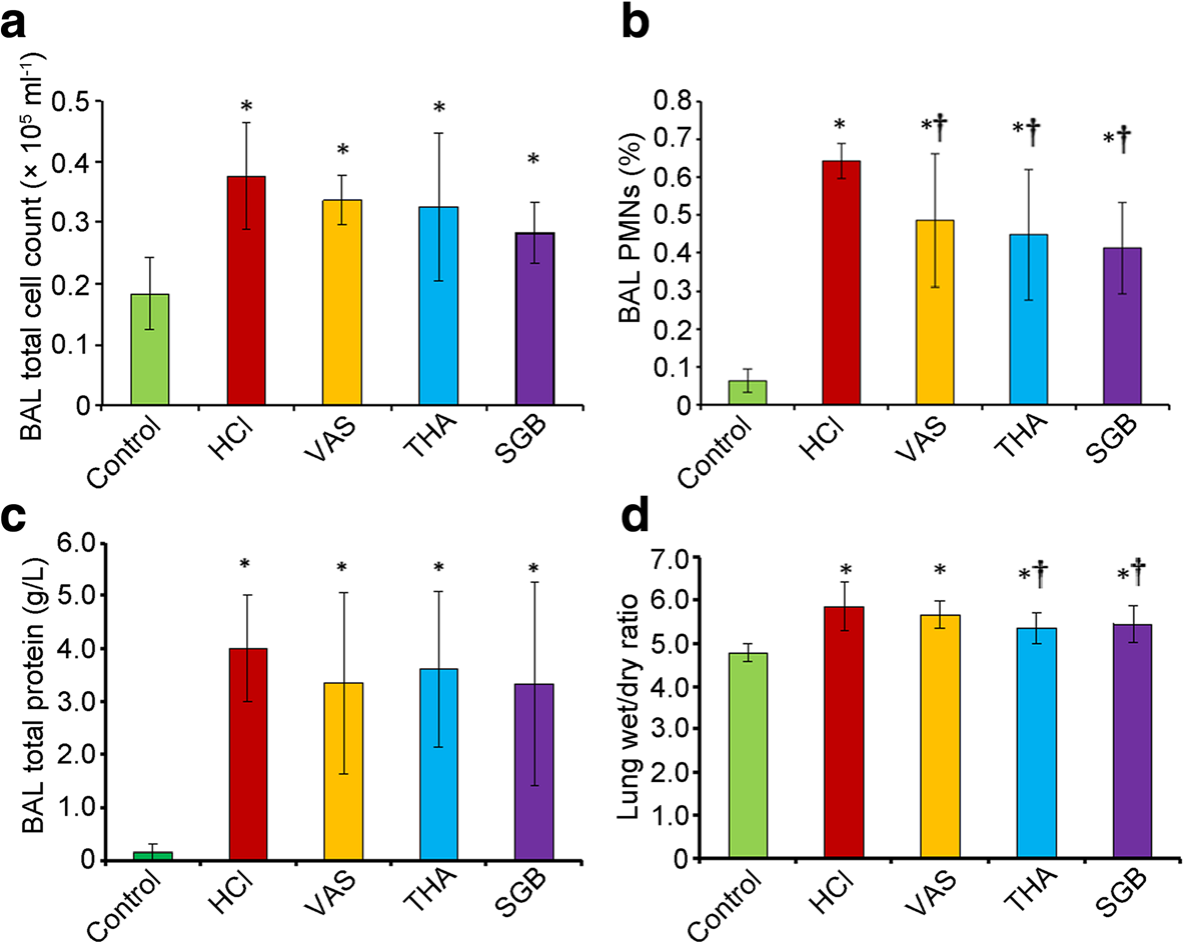
Inflammatory cell infiltration and vascular permeability changes induced by HCl aspiration in groups with different treatments. **a**, BAL total cell count; **b**, BAL PMNs (%); **c**, BAL total protein; **d**, lung wet/dry ratio. For control group (*n* = 8), rabbits only received saline intratracheally injection (2.8 ml/kg) after surgery; for HCl groups (*n* = 8), ALI model was established by intratracheal injection of HCl (pH 1.5, 2.8 ml/kg); for VNS groups (*n* = 8), after the ALI model established by HCl, rabbits also received constant voltage stimuli (5 V, 2 ms, and 1 Hz); for THA group (*n* = 8), after ALI model established by HCl, rabbits also received THA (3 mg/kg) intravenous injection; and for SGB group, after ALI model established by HCl, rabbits also continuously received 0.25% bupivacaine pumping via epidural catheter (3 mg/kg). BAL, bronchoalveolar lavage; PMNs, polymorphonuclear neutrophils; VNS, vagus nerve stimulation; THA, tetrahydroaminoacridine; SGB, stellate ganglion block; * *P* < 0.05, compared to the control group; † *P* < 0.05, compared to the HCl group

As shown in Fig. 4, HCl aspiration significantly increased the BALF total protein level, leukocyte count, percentage of PMNs and the W/D ratio when compared to the control group (t = 0.19, 0.58, 5.47 and 5.49, *P* = 0.000, 0.000, 0.000 and 0.000, respectively). All three treatments could alleviate the changes induced by HCl, but the attenuating effects by three treatments in BALF total protein level (Fig. 4a) and leukocyte count (Fig. 4c) were not significant. But treatments of VAS, THA and SGB could significantly decrease the elevated percentage of PMNs (t = 0.16, 0.19 and 0.23, *P* = 0.017, 0.004 and 0.001, respectively) (Fig. 4b), and THA and SGB could significant reduce the improvement of lung wet/dry ratio (t = 2.59 and 2.10, *P* = 0.014 and 0.043) (Fig. 4d) induced by HCl inspiration.

### Changes in inflammatory factors in both BALF and plasma

**Fig. 5:**
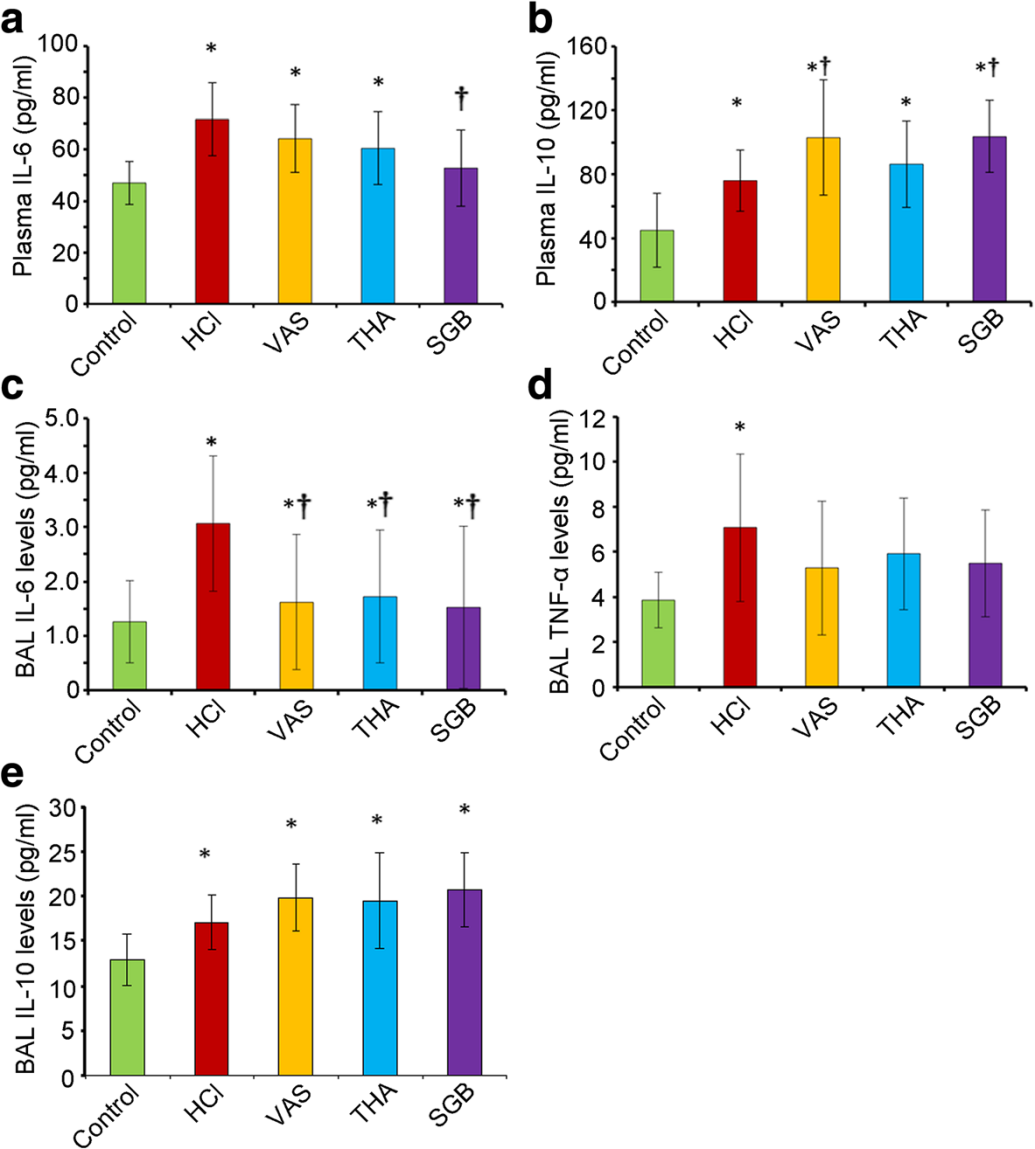
Changes of inflammatory factors in both BALF and plasma in rabbits with acute lung injury after three treatments. **a**, plasama IL-6 levels; **b**, plasama IL-10 levels; **c**, BAL IL-6 levels; **d**, BAL TNF-α levels; **e**, BAL IL-10 levels. For control group (*n* = 8), rabbits only received saline intratracheally injection (2.8 ml/kg) after surgery; for HCl groups (*n* = 8), ALI model was established by intratracheal injection of HCl (pH 1.5, 2.8 ml/kg); for VNS groups (*n* = 8), after the ALI model established by HCl, rabbits also received constant voltage stimuli (5 V, 2 ms, and 1 Hz); for THA group (*n* = 8), after ALI model established by HCl, rabbits also received THA (3 mg/kg) intravenous injection; and for SGB group, after ALI model established by HCl, rabbits also continuously received 0.25% bupivacaine pumping via epidural catheter (3 mg/kg). BALF, bronchoalveolar lavage fluid; VNS, vagus nerve stimulation; THA, tetrahydroaminoacridine; SGB, stellate ganglion block; * *P* < 0.05, compared to the control group; † *P* < 0.05, compared to the HCl group

In the HCl group, significant increase in IL-6 (Fig. 5a) and IL-10 level (Fig. 5b) in plasma was found compared to those in control group (t = 24.65 and 31.17, *P* = 0.000 and 0.000). All three treatments attenuated these changes induce by HCl aspiration, but only SGB in plasma IL-6 (t = 18.88, *P* = 0.007), and VNS and SGB in plasma IL-10 (t = 26.99 and 27.59, *P* = 0.047 and 0.043) were significant while compared with the HCl group. Meanwhile, an obvious increase of IL-6 level in BALF was also identified in HCl group when compared to the control group (t = 1.80, *P* = 0.005), but this elevation was significantly decreased in VAS, THA and SGB group (t = 1.39, 1.34, and 1.42; *P* = 0.021, 0.031 and 0.014, respectively) (Fig. 5c). Similarly, a remarkably improvement of TNF-α was also detected after treated with HCl (t = 3.21, *P* = 0.017), but none of three treatments could significantly attenuate this improvement and none significant difference was examined between control and three treated groups (Fig. 5d). In addition, IL-10 level in BAFL was also upregulated in HCl group while compared with control group, but after treatment, the levels of IL-10 in BALF in VNS, THA and SGB group were even higher than that in HCl group, but no significant differences were identified (Fig. 5e).

### Histological changes of lung tissues after different treatment

**Fig. 6:**
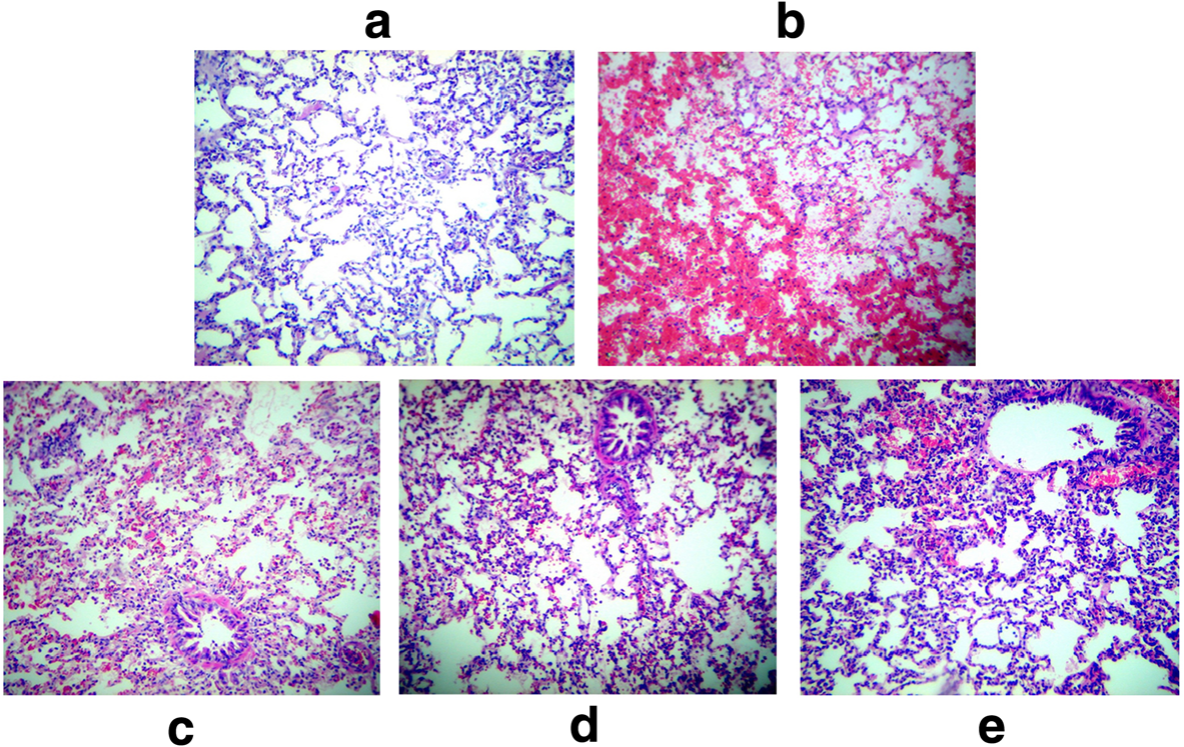
Histological changes of lung tissues detected by light microscope at 200×. **a**, control group; **b**, HCl group; **c**, vagus nerve stimulation (VNS) group; **d**, tetrahydroaminoacridine (THA) group and **e**, stellate ganglion block (SGB) group. For control group (*n* = 8), rabbits only received saline intratracheally injection (2.8 ml/kg) after surgery; for HCl groups (*n* = 8), ALI model was established by intratracheal injection of HCl (pH 1.5, 2.8 ml/kg); for VNS groups (*n* = 8), after the ALI model established by HCl, rabbits also received constant voltage stimuli (5 V, 2 ms, and 1 Hz); for THA group (*n* = 8), after ALI model established by HCl, rabbits also received THA (3 mg/kg) intravenous injection; and for SGB group, after ALI model established by HCl, rabbits also continuously received 0.25% bupivacaine pumping via epidural catheter (3 mg/kg)

**Fig. 7:**
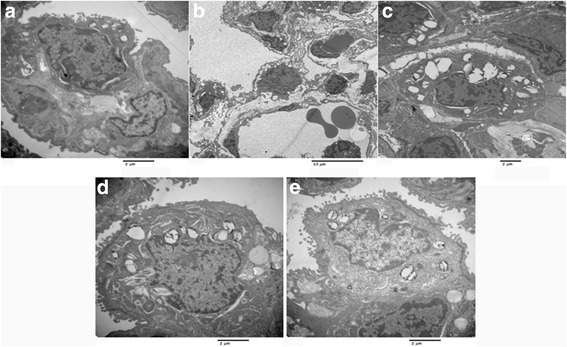
Histological changes of lung tissues detected by electron microscopy. **a**, control group; **b**, HCl group; **c**, vagus nerve stimulation (VNS) group; **d**, tetrahydroaminoacridine (THA) group and **e**, stellate ganglion block (SGB) group. For control group (*n* = 8), rabbits only received saline intratracheally injection (2.8 ml/kg) after surgery; for HCl groups (*n* = 8), ALI model was established by intratracheal injection of HCl (pH 1.5, 2.8 ml/kg); for VNS groups (*n* = 8), after the ALI model established by HCl, rabbits also received constant voltage stimuli (5 V, 2 ms, and 1 Hz); for THA group (*n* = 8), after ALI model established by HCl, rabbits also received THA (3 mg/kg) intravenous injection; and for SGB group, after ALI model established by HCl, rabbits also continuously received 0.25% bupivacaine pumping via epidural catheter (3 mg/kg)

Compared to the control group, lung histology with considerable hemorrhage, edema, consolidation, atelectasis and neutrophil infiltration was found in lung tissue sections of rabbits in the HCl group under the light microscope (Fig. 6). Under electron microscopy, several pathological changes including swelling of alveolar epithelial type I cell and microvascular endothelial cell, cavitation of mitochondria, reduction of mitochondrial cristae, vacuolation of the lamellar body of alveolar epithelial type II cells, shedding of microvilli, and basilar membrane rarefaction were observed in lung tissues compared HCl group with control group (Fig. 7). All three treatments could remarkably alleviate these pathological changes induced by HCl aspiration (Figs. 6 and 7).

## Discussion

Several conditions such as trauma, shock and stress can trigger SNS hyperactivity, leading to excessive release of epinephrine and norepinephrine. Increase in epinephrine and norepinephrine level in plasma causes pulmonary vasoconstriction and capillary hyperpermeability, reduces pulmonary surfactant activity, and further induces pulmonary arteriolar remodeling and promotes the formation of pulmonary arterial hypertension [10, 11]. We performed the present study through the establishment of an ALI model induced by HCl aspiration in rabbits.

In this study, SNS and PNS activity was assessed by monitoring the changes of several indicators including HRV, CSTDF and CVTDF. After HCl aspiration, both SNS and VNS were activated, while ANS homeostasis was shifted towards the SNS confirmed by the significant increase of CVTDF and CSTDF. In company with the activation of sympathetic and parasympathetic activity, LF (nu) and LF/HF significantly increased and HF (nu) significantly reduced in HRV analysis. The LF/HF ratio is considered to reflect sympathovagal balance [12]. This imbalance can be restored by VNS and THA treatment via activating CAP and by SGB via inhibiting sympathetic hyperactivity, demonstrated by more stable fluctuation of LF, HF and LF/HF curves after all three treatments. Lung injury and systemic inflammatory response in rabbits with HCl-induced ALI were alleviated, with improvement of pulmonary function.

Our study showed that SGB can regulate ANS balance by inhibiting excessive SNS activity and effectively alleviate nociceptive responses, which is consistent with previous description [3, 13]. In addition, rabbits in the SGB group achieved the most improvement from HCl aspiration in our study, with no obvious effects on HR and MAP throughout the experiment. However, a local anesthetic (bupivacaine) was given to animals after surgical exposure of the stellate ganglion in the SGB group, which might lead to local diffusion of anesthetic though the cervical incision and/or spreading to surrounding areas adjacent to the vagus nerve.

The most important function of PNS is supposed to be executed by the vagus nerve, and the anti-inflammatory effect of the efferent vagus nerve is widely known as the activation of the CAP now [14, 15]. Tracey et al. thought that the tonic activity of the vagus nerve is essential for maintaining immune homeostasis [15]. Ma P et al. suggested that strengthen of vagal nerve activity can attenuate the systemic inflammatory response to endotoxin [14]. THA has central cholinomimetic properties, and as a potent acetylcholinesterase inhibitor, has been used for the treatment of Alzheimer’s disease [16, 17]. Previous studies indicated that central or intravenous injection of THA could reverse hypotension and decrease systemic TNF-α response [7, 18]. In the present study, intravenous infusion of THA in rabbits with HCl-induced ALI displayed similar effects with VNS on activating PNS activity, inhibiting systemic and local inflammatory responses, and improving pulmonary function. Additionally, it is apparent that intravenous injection of THA is more convenient than injection of VNS in clinical application.

After noxious stimulation, the discharge frequency of cervical afferent and efferent vagus nerve both increased remarkably as described previously [19, 20]. In our study, the CSTDF and CVTDF were significantly increased within the first 5 min after HCl aspiration, and the highest average value lasted over 1 h in sympathetic nerves and over 2 h in the vagus nerve. In addition, CVTDF in three treatment groups was all lower than the HCl group, which was even lower than the control group. It might be explained that HCl aspiration can induce elevation of both afferent and efferent cervical vagal nerve discharge, while the afferent vagal nerve discharge may play a predominant role in the discharge frequency of intact cervical vagus trunk [21]. With an increase in PNS activity triggered by three treatments directly or indirectly, the efferent vagus nerve activity became more active, leading to a decrease of the absolute value of CVTDF even lower than the control group.

Signals for inflammatory mediator release of brain are via the circulation or afferent fibers of the vagus nerve, thus SNS and/or PNS activity is activated. Sloan et al. have documented that IL-6 levels were inversely related with vagus nerve activity indexed by HF-HRV [22]. Meanwhile, Weber et al. have reported that low HRV can delay the recovery of TNF-α after stressor ending compared with ones with high HRV [23]. In addition, Tonhajzerova et al. demonstrated that IL-6 is an independent risk factor for cardiovascular mortality [24]. In our study, the pro-inflammatory factors IL-6 and TNF-α in the plasma and BALF markedly decreased and the anti-inflammatory factor IL-10 was further increased in rabbits with ALI after three treatments. These results indicated that an obvious improvement of a systemic and local inflammatory response was obtained after regulating homeostasis of the ANS by THA, VNS and SGB.

## Conclusion

In summary, we have gained following based on our results: firstly, both SNS and PNS activity were markedly elevated in rabbits with HCl-induced ALI and the disturbance of ANS homeostasis was attributed to a predominance of SNS activity; Secondly, all three treatment methods including VNS, THA and SGB could effectively regulate the disequilibrium of ANS, and improve pulmonary function; thirdly, SGB treatment was the most effective approach in regulating ANS balance. However, further preclinical and clinical studies are still required to further explore the efficacy and safety of these approaches in patients with inflammatory disorders, such as aspiration pneumonia.
